# Evaluation of Patient’s Quality of Life before and after Implantation of Abbott’s Proclaim™ XR Spinal Cord Stimulator with BurstDR™ Stimulation in Chronic Pain Syndrome

**DOI:** 10.3390/medicina59122192

**Published:** 2023-12-17

**Authors:** Wojciech Tomasz Ślusarczyk, Tadeusz Jerzy Nejman, Maciej Laskowski, Agnieszka Koperczak, Agnieszka Stanuszek, Marcin Ciekalski

**Affiliations:** Department of Neurosurgery, University Clinical Center, Faculty of Medical Sciences in Katowice, Medical University of Silesia, 40-752 Katowice, Poland; tdn.nch@gmail.com (T.J.N.);

**Keywords:** SCS (spinal cord stimulation), FBSS (failed back surgery syndrome), BurstDR™, quality of life after SCS, analgesics after SCS

## Abstract

*Background and Objectives:* In case of the ineffectiveness of pharmacological and non-pharmacological treatments in managing chronic neuropathic pain, spinal cord stimulation (SCS) with BurstDR™ stimulation may reduce pain and increase the quality of life. The term “burst” refers to a series of stimulation impulses that are compressed into small packets and separated by intervals of latency. *Materials and Methods*: A group of 30 consecutive patients who received the BurstDR™ stimulator using the minimally invasive percutaneous method was selected. Patients selected for our study underwent numerous spinal surgeries before SCS implantation. In the study, analgesics and co-analgesics and their doses used by patients before and 6 months after SCS implantation were examined and compared. Using the visual analogue scale (VAS), pain was compared before and after the procedure. Patients` quality of life was assessed using the Oswestry Disability Index (ODI). *Results:* We observed a significant reduction in opioid daily doses by an average of 32.4% (±36.1%) and a reduction in paracetamol daily doses by an average of 40% (±33.4%). There was a reduction in pregabalin doses as well. Ketoprofen daily dose reduction was 85.4 mg. The mean VAS difference before and after procedure was 3.9 (±2.3), and the mean difference in ODI was 12.9 (±9), which benefits operative treatment. The VAS and ODI results were statistically significant as well. *Conclusions:* According to our research, BurstDR™ stimulation improves the quality of life by reducing doses of analgesics and the level of pain.

## 1. Introduction

The International Association for the Study of Pain (IASP) defines pain as an unpleasant sensory and emotional experience associated with actual or potential tissue damage [1]. Chronic pain affects more than 30% of people worldwide and imposes a significant personal and financial burden [2]. According to the Pain Foundation in the United States, chronic pain is the leading reason for seeking medical help and costs the U.S. approximately $635 billion each year in healthcare expenses, disability, and lost productivity [3]. Many surgical procedures carry significant postoperative chronic pain complications [4], and it is worth noting that chronic post-surgical pain affects up to 10% of patients [5].

Pain is a multidimensional human experience with sensory, affective, and cognitive components. These components provide information about the location, timing, and character of a stimulus that triggers pain and elicit feelings and responses to that stimulus. These three elements combine to form the pain experience during a painful episode [6]. Based on the pathophysiological mechanisms, duration, anatomical location, and the presence of malignancy, pain can be categorized into different types. Understanding each classification and its definition is crucial for guiding diagnosis [7]. Pain duration distinguishes pain as either acute or chronic. Acute pain develops suddenly after an injury and is typically very severe. Pain that persists for longer than three months is categorized as chronic [8]. Based on the pathophysiological mechanisms, pain can be classified as nociceptive or neuropathic. Nociceptive pain arises from tissue damage that activates pain receptors known as nociceptors. Neuropathic pain, on the other hand, is caused by damage to the nerve cells in the peripheral or central nervous system [9]. Neuropathic pain is a condition involving both molecular and structural changes in the central nervous system (CNS). Therefore, distinguishing between nociceptive and neuropathic pain is critical, as these pain types usually require different treatment approaches. Recently, there is also a growing interest among pain societies focused on a “new”, third type of pain called nociplastic. Its mechanism is not entirely understood, but it is suggested that augmented CNS pain, sensory processing, and altered pain modulation may be to blame. The pain is multifocal, more widespread or intense, and may co-exist with nociceptive or neuropathic pain, for example in low back pain [10].

The lower back, which anatomically extends from the 12th rib to the iliac crest, often co-exists with and is mistaken for buttock pain. It encompasses various types of pain, including nociceptive pain, neuropathic (radicular) pain that radiates down the legs, and occasionally, nociplastic pain, which results from amplified pain signals within the central nervous system [11]. The pathogenesis of lower back pain is influenced by a variety of causes and risk factors, such as disc degeneration, radicular pain, facet arthropathy, myofascial pain, sacroiliac joint pain, spondyloarthropathies, and nociplastic pain [11].

A wide range of pharmacological and non-pharmacological treatments have been employed to manage lower back pain. According to the guidelines of the American College of Physicians, non-steroidal anti-inflammatory drugs (NSAIDs) or muscle relaxants are considered the first-line treatment for acute or subacute lower back pain. Tramadol or duloxetine are suggested as second-line treatments for chronic lower back pain, with opioids being considered a last option [12]. Nevertheless, they remain the most effective analgesics and have been used to manage pain for over a thousand years. However, they carry serious side effects, including addiction, dependence, constipation, sedation, respiratory depression, immunosuppression, and neuro-hormonal dysregulation. The use of opioids to treat chronic non-malignant pain is currently a subject of intense debate due to those side effects and the risk of potential overdose [6]. In recent years, the drugs involved in overdose deaths have changed. Fatality rates related to drug overdoses involving synthetic opioids (such as fentanyl, fentanyl analogs, and tramadol) other than methadone increased by 45%, rising from 6.2 per 100,000 in 2016 to 9.0 in 2017 in the United States [13].

Pharmacological therapies are limited and often associated with numerous side effects. However, there are emerging pain management pathways, such as neuromodulation, that do not entail the systemic side effects of pharmacotherapies. The International Neuromodulation Society defines neuromodulation as “the alteration of nerve activity through the targeted delivery of a stimulus, such as electrical stimulation or chemical agents, to specific neurological sites in the body” [14]. Neuromodulation is a rapidly expanding field of pain medicine, encompassing a range of non-invasive, minimally invasive, and surgical electrical therapies.

The use of electrical stimulation for modern pain management began in 1967, inspired by Melzack and Wall’s gate control theory. This theory is based on the concept that the stimulation of fast-velocity mechanoreceptive Aβ fibers can block nociceptive signals transmitted by Aδ and C fibers from reaching higher brain centers, resulting in analgesia [15]. Neuromodulation therapies include deep brain and motor cortex stimulation, peripheral nerve stimulation, as well as non-invasive treatments like repetitive transcranial magnetic stimulation, transcranial direct current stimulation, transcutaneous electrical nerve stimulation, and spinal cord stimulation (SCS) [6].

For chronic neuropathic pain, the most common neuromodulation treatment is spinal cord stimulation [16]. SCS involves the placement of a subcutaneous implantable pulse generator connected to leads that traverse into the epidural space posterior to the spinal cord dorsal columns. The mechanisms through which neurostimulation inhibits pain include gating dorsal horn neurons through the activation of Aβ fibers and inhibitory interneurons [14].

Several novel stimulation modalities, including tonic, high-frequency, burst, dorsal root ganglion (DRG) stimulation, and other paradigms, have been introduced over the past few years [17]. The fundamental type of stimulation is tonic stimulation (low frequency, LF), which uses a frequency of 60–120 Hz. High-frequency stimulation (high frequency, HF) uses frequencies ranging from 3000 Hz to 10,000 Hz, and it provides better analgesic effects compared to the tonic stimulation.

It is important to provide a clear definition of “burst” stimulation. De Ridder et al. introduced a novel waveform known as “burst” in 2010, which has since been patented and renamed Burst-DR (Abbott, Plano, TX, USA). This waveform is primarily used in SCS to treat back and leg pain [18]. The term “burst” refers to a series of stimulation impulses that are condensed into small packets and separated by periods of dormancy. According to De Ridder`s definition of burst (D-Burst), bursts consist of five separate 1000-millisecond spikes separated by passive recharge intervals ranging from 4 to 1000 milliseconds. In this particular version, each spike interacts synergistically with the preceding one, resulting in a gradual plateau in calcium influx that ultimately leads to charge accumulation. The passive dissipation of this charge accumulation produces a nonlinear “super action potential” larger than the sum of all individual spikes, followed by a quiescent phase or dormancy before the next packet begins [16,19,20]. It is believed that BurstDR recruits groups of neurons with three important functions: increasing postsynaptic responses to presynaptic action potentials, strengthening synaptic connectivity, and activating parallel, integrated anatomical pathways [21].

The aim of our study was to evaluate the effectiveness of Abbott’s Proclaim XR Spinal Cord Stimulator with BurstDR™ stimulation in patients with chronic low back or limb pain. As determinants in our assessment, we used the visual analogue pain scale (VAS), Oswestry Disability Index (ODI), and changes in doses of pain medications taken by the patients before and 6 months after stimulation implantation.

## 2. Materials and Methods

This retrospective trial was conducted at a neurosurgical clinic within an academic medical center. The analysis involved a group of 30 consecutive patients who underwent SCS implantation in our department from January 2021 to March 2023. This group received one of the most modern SCS systems, the BurstDR™, implanted minimally invasively solely through the skin. The study included adult patients, consisting of 53.3% men and 46.7% women, with an average age of 56 years. All participants were qualified for SCS due to their chronic lower back issues persisting for many years. Inclusion criteria for the SCS trial were: age ≥ 18 years, with chronic pain lasting 6 months or more, and failure of medical management or other more conservative treatment modalities. Typical exclusion criteria included coagulopathy, systemic or local infections, and use of pacemakers. There was no control group.

Before qualification for SCS, the patients we treated in the Pain Clinic with no satisfactory results underwent numerous courses of rehabilitation and physiotherapy and underwent psychological consultation to exclude somatization. All patients underwent exactly the same surgical treatment always performed by the same experienced surgeons (two neurosurgeons). SCS implantation surgery can be divided into three main stages consisting of electrode implantation, intraoperative test stimulation, and implantation of the stimulator after 14 days. The electrode is implanted subcutaneously under the vertebral arch into the spinal canal, with prior verification of the location by saline injection, to the previously determined level. The electrode is placed at the dorsal medial surface of the dural sac under the X-ray. As the electrode implantation process is completed, the intraoperative test stimulation begins by using tonic stimulation to verify the correct placement of the electrode. When surgeons operate using local anesthesia, they can communicate verbally with the patient. This interaction helps to confirm the right electrode placement, as the patient can describe where they feel sensations during tonic stimulation. Subsequently, the electrode connector is led out and connected to the external stimulator used by the patient for the following 7 days to manually modulate the stimulation intensity. After 14 days have passed, removal of the connector is performed, and the subcutaneous SCS stimulator is placed and stitched into the subcutaneous pocket. All patients received rehabilitation treatment after the surgery as a part of the postoperative protocol.

In the study, we examined analgesics (paracetamol, ketoprofen, tramadol, morphine, codeine, oxycodone, and tapentadol) and co-analgesics (pregabalin), along with their daily doses, used daily by the patients before and 6 months after SCS implantation. The medications were used as needed before the surgery and afterwards. The doses were self-reported by the patients, who helped us calculate the average daily dose a whole month before the procedure and then, similarly, 6 months after SCS implantation. We noted those doses of drugs upon admission to the hospital for electrode placement and then again six months after the surgery. The difference between the doses was calculated and expressed in percents of initial preoperative dose. Opioid data were quantified in morphine milligram equivalents (MEq). The primary outcome measure focused on pain intensity, which was assessed using a visual analogue scale (VAS). We chose the scale, as it was the easiest for patients. Additional data collected included pain-related disability measured using the Oswestry Disability Index (ODI). The data were gathered from patients during interviews before and 6 months after the surgery and noted on special questionnaires.

Methodology and procedures were performed in accordance with the ethical standards established by the 1964 Declaration of Helsinki and its subsequent amendments. We obtained informed consent from all the patients before study inclusion.

Statistical analysis of the collected data was conducted using the Statistica 13.0 software (StatSoft, Krakow, Poland). Categorical variables were described using numbers and percentages, while quantitative variables were described using the mean and its confidence interval or median. The normality of the distribution of the obtained results was assessed using the Shapiro–Wilk test. The changes in pain intensity and quality of life before and 6 months after intervention were assessed with a paired *t*-test or Wilcoxon signed-rank test, depending on the normality of distribution.

## 3. Results

The aim of our study was to assess the effectiveness of Abbott’s Proclaim XR Spinal Cord Stimulator with BurstDR™ stimulation. One of the determinants chosen was the changes in doses of pain medications taken by the patients before and 6 months after stimulation implantation. We observed a reduction in opioid daily doses by an average of 32.4% (±36.1%). A statistically significant difference prior to and after the SCS implantation was demonstrated (*p* = 0.016) (Table 1 and Table 2).

Paracetamol daily dose reduction reached an average of 40% (±33.4%). However, no statistically significant difference was observed regarding the paracetamol daily dose (*p* = 0.1) (Table 3).

Before the surgery, six patients received an average pregabalin dose of 350 mg, which increased to an average dose of 431 mg for four patients after surgery. Two out of six patients taking it before stopped taking the drug completely. Another two had their dose reduced by more than 50%. Only one patient had an increase in the dose of the drug, but his dose of opioids was reduced (Table 4).

The mean reduction in ketoprofen dosage was 85.4 mg. Unfortunately, there were not enough patients to perform statistical analysis for co-analgesics and the non-steroid anti-inflammatory drugs NSAID (Table 5). Based on our data, only the tendency to reduce the doses of pregabalin and ketoprofen 6 months after the surgery is visible.

Other determinants used in the evaluation of the effectiveness of Abbott’s Proclaim XR Spinal Cord Stimulator with BurstDR™ stimulation in patients with chronic low back or limb pain were assessing the visual analogue pain scale (VAS) and assessing the Oswestry Disability Index (ODI) before and 6 months after stimulation implantation.

**Figure 1:**
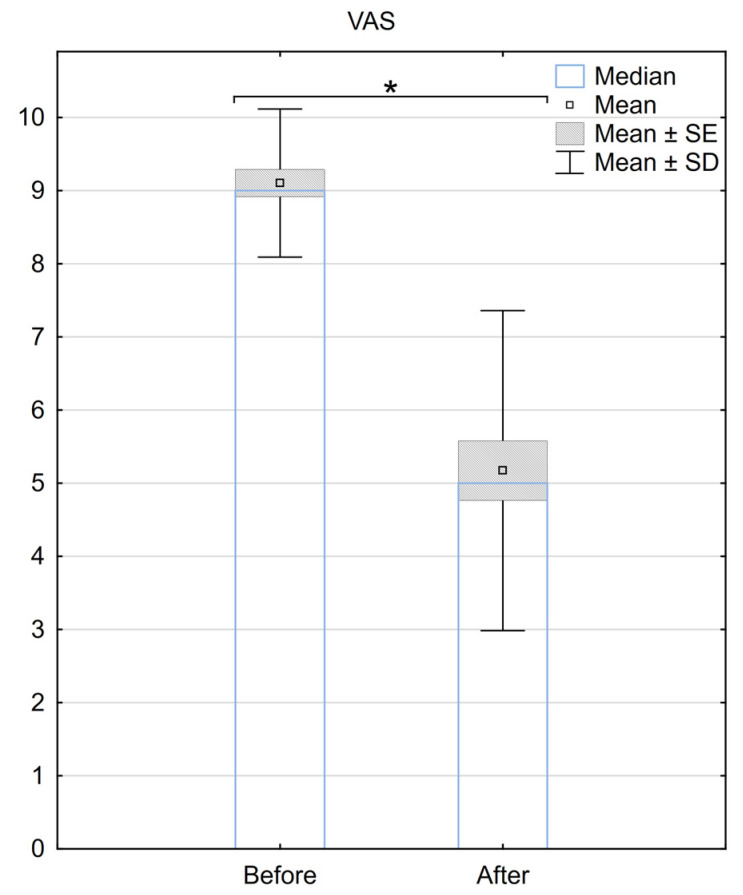
Reduction in pain according to the VAS scale 6 months after the implantation of BurstDR™. SE—standard error, SD—standard deviation, * Indicates statistical significance at a significance level of *p* < 0.05.

The median VAS score before the surgery was 9 (±0.97), and it decreased to 5 (±2.23) after the SCS implantation. The mean difference in the VAS scale before and after the procedure was 3.9 (±2.3), with a median difference of 4. A statistically significant difference between the two groups was demonstrated (*p* < 0.001) (Figure 1).

**Figure 2:**
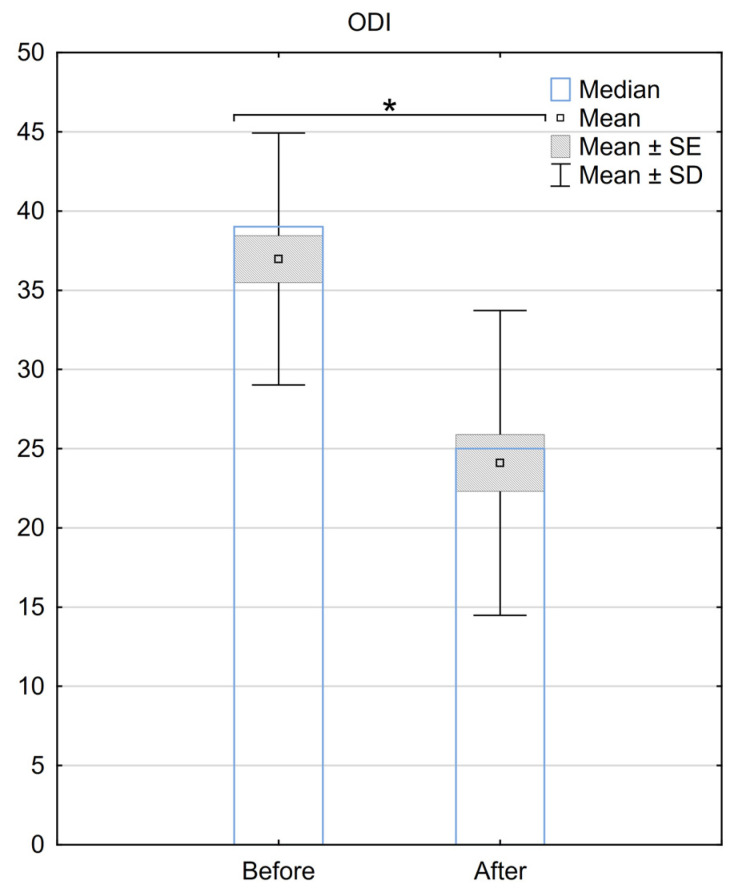
Reduction in the ODI scale 6 months after the implantation of BurstDR™. SE—standard error, SD—standard deviation, * Indicates statistical significance at a significance level of *p* < 0.05.

Regarding the ODI, the median score before the procedure was 39 (±7.2), and it improved to 28 (±9.8) after the surgery. The mean difference in ODI before and after the procedure was 12.9 (±9), with a median difference of 11. A statistically significant difference between ODI scores for the two groups was demonstrated (*p* < 0.001) (Figure 2.).

Over 90% of patients claimed that they would agree to the implantation of SCS with BurstDR™ stimulation again to treat their pain.

## 4. Discussion

Our study demonstrated the effectiveness of BurstDR™ therapy for 30 patients with failed back surgery syndrome (FBSS) who underwent a standardized two-stage procedure. All of these patients suffered from neuropathic chronic back and/or lower limb pain. We achieved significant and consistent pain relief for both back and lower limb pain. Additionally, differences in VAS and ODI scores indicated a significant improvement in patients’ quality of daily life. Our results are in line with the paper of Karri et al., who identified 11 studies that included SCS waveform comparisons for treating chronic lower back pain primarily caused by FBSS [22]. A pooled meta-analysis of five studies comparing tonic waveforms and BurstDR™ stimulation found that BurstDR stimulation significantly reduced pain scores. It was also observed that the BurstDR stimulation waveform was the first to establish level 1A evidence for chronic low back pain [22].

Another study involving BurstDR, conducted by Hunter et al., categorized patients into two primary cohorts based on whether BurstDR stimulation was enabled through surgical revision to a new system (DR-S) or by a programming change to an existing system (DR-ON) [16]. Following BurstDR™ stimulation, 80% of patients who previously expressed dissatisfaction with their SCS system reported satisfaction one year later. After transitioning to BurstDR™ stimulation, the average percentage of pain relief in the patient group increased from 34% to 58%. Additionally, patients who received BurstDR™ stimulation reduced their overall opioid consumption by 25% [16]. Our data also confirm a reduction in opioid usage by over 32.4%. This is particularly important, given the fact that long-term high doses of opioids for chronic pain have been associated with negative health impacts and societal costs. The results obtained with BurstDR™ therapy are consistent with studies that utilized BurstDR™ spinal cord stimulation for FBSS [16,22].

Furthermore, a prospective study conducted by Mons MR et al. found that the BurstDR™ Spinal Cord Stimulation in non-operated discogenic low back pain disease resulted in a significant reduction in back pain, leg pain, and quality of life in patients, decreasing the level of disability rated in ODI and quality of life, which persisted during the 12 months of the study [23]. It is an important observation that cannot be compared to our group, as we qualified for SCS only the patients who had their spine operated on before.

Moreover, it should be mentioned that the number of patients taking paracetamol increased in our study after 6 months of observation, despite the reduction in the mean daily dose. Before the surgery, five patients took paracetamol, with a mean dose of 1570 mg (±878 mg). After the surgery, nine patients used paracetamol, with a mean dose of 866.7 mg (±460 mg). In that group, we observed daily dose reduction by an average of 40% (±33.4%), but because of the enlargement of the group, the result was statistically insignificant (*p* = 0.1). However, this does not indicate the ineffectiveness of the treatment because the patients who started taking paracetamol discontinued or significantly reduced the doses of opioid drugs. This is associated with the quality of life`s improvement through the reduction in the opioids’ side effects. A common positive change reported by patients was the ability to drive a car again.

Discussing the anesthesia and the lead placement during the first stage of the procedure, we performed it under local anesthesia with intraoperative tonic stimulation for optimal electrode placement and optimal coverage of the pain area. However, both a prospective randomized multicenter study conducted by Pope J.E. et al. [24] and the CRISP study [25] proved that there is no difference between leads placed with anatomical placement (AP) and leads placed based on paresthesia mapping (PM) when using BurstDR™ stimulation. That prompts a change in the method of implantation in the future.

Our study confirms the effectiveness of BurstDR™ stimulation and validates its consideration in low back and leg pain therapy.

There were certain limitations in our research that warrant further discussion and investigation. The good results obtained in the study were certainly influenced by the appropriate qualification of patients for the SCS procedure. Additionally, final implantation was performed only in patients who had a good response to trial stimulation, which lasted for 7 days. People who underwent the electrode removal after the stimulation trial period were not included in the study, due to the lack of improvement in the reported symptoms. Moreover, there was no control group in our study. We did not analyze other co-founding factors or patients’ general medical conditions either. Finally, our sample size was rather small, which is a standard problem in single-center studies.

## 5. Conclusions

In our analysis, burst-type SCS stimulation proved to be an effective method for treating chronic pain. Its implantation resulted in a reduction in the doses of analgesics, especially opioids, which are the most addictive and dangerous when overdosed. The reduction in pain medication intake was associated with the mean VAS score decreasing by 4 six months after the surgery. Additionally, the ODI score decreased by 11 after the procedure. Thanks to the minimally invasive implantation method performed under local anesthesia, the procedure is safe, even for individuals with disabilities. The ability to perform stimulation during the procedure ensures the precise placement of the electrode, providing full coverage of the painful areas, which contributes to the high effectiveness of stimulation after the procedure.

## Figures and Tables

**Table 1:** Opioids and its doses taken by the patients before and 6 months after the procedure.

Opioid	N Before	% Before	Average Dose Before	Std. Dev.	N After	% After	Average Dose After	±
Tramadol	22	84.6	218.2	141.5	12	85.8	134.2	80.1
Morphine	1	3.8	300	-	1	7.1	150	-
Codeine	1	3.8	300	-	0	-	-	-
Oxycodone	1	3.8	140	-	1	7.1	60	-
Tapentadol	1	3.8	125	-	0	-	-	-

N—number of patients taking particular medications. Before—before SCS implantation. After—control 6 months after SCS implantation.

**Table 2:** Opioids data given in morphine milligram equivalents.

Opioids Together	N Before	% Before	Average Dose Before	Std. Dev.	N After	% After	Average Dose After	±
	26	100%	41.2	65.8	14	100%	28.9	41

N—number of patients taking particular medications. Before—before SCS implantation. After—control 6 months after SCS implantation.

**Table 3:** Paracetamol doses before and 6 months after the procedure.

Non-Opioid Analgesic	N Before	% Before	Average Dose Before	Std.Dev.	N After	% After	Average Dose After	±
Paracetamol	5	100%	1570	878	9	100%	866.7	460

N—number of patients taking particular medications. Before—before SCS implantation. After—control 6 months after SCS implantation.

**Table 4:** Pregabalin doses before and 6 months after the procedure.

Co-Analgesic	N Before	% Before	Average Dose Before	From. Std.	N After	% After	Average Dose After	±
Pregabalin	6	100%	350	324	4	100%	431	712

N—number of patients taking particular medications. Before—before SCS implantation. After—control 6 months after SCS implantation.

**Table 5:** Ketoprofen doses before and 6 months after the procedure.

NSAIDs	N Before	% Before	Average Dose Before	From. Std.	N After	% After	Average Dose After	±
Ketoprofen	8	100%	268.75	92.34	5	100%	186	152.4

N—number of patients taking particular medications. Before—before SCS implantation. After—control 6 months after SCS implantation.

## Data Availability

No data in repository.
